# Effect of dietary supplementation with *Spirulina on* the expressions of *AANAT*, *ADRB3*, *BTG2 and FASN* genes in the subcutaneous adipose and *Longissimus dorsi* muscle tissues of purebred and crossbred Australian sheep

**DOI:** 10.1186/s40781-015-0047-3

**Published:** 2015-03-04

**Authors:** Arash Kashani, Benjamin William Behrens Holman, Peter David Nichols, Aduli Enoch Othniel Malau-Aduli

**Affiliations:** Animal Science and Genetics, Tasmanian Institute of Agriculture, School of Land and Food, Faculty of Science, Engineering and Technology, University of Tasmania, Private Bag 54 Sandy Bay, Hobart, Tasmania 7001 Australia; New South Wales Department of Primary Industries, Centre for Red Meat and Sheep Development, Cowra, 2794 New South Wales Australia; CSIRO Food and Nutrition, Oceans and Atmosphere Flagships, Hobart, TAS 7001, Australia; Veterinary and Biomedical Sciences, College of Public Health, Medical and Veterinary Sciences, Division of Tropical Health and Medicine, James Cook University, Townsville, Queensland 4811 Australia

## Abstract

**Background:**

The demand for healthy, lean and consistent meat products containing low saturated fatty acid content and high quality polyunsaturated fatty acids (PUFA), especially long-chain (≥C_20_) omega-3 PUFA, has increased in recent times. Fat deposition is altered by both the genetic background and dietary supplements, and this study aimed to assess the effect of dietary *Spirulina* supplementation levels on the mRNA expression patterns of genes controlling lipid metabolism in the subcutaneous adipose tissue (SAT) and *Longissimus dorsi* (*ld*) muscle of Australian crossbred sheep.

**Methods:**

Twenty-four weaned lambs belonging to four breeds under the same management conditions were maintained on ryegrass pasture and fed three levels of *Spirulina* supplement (control, low and high). In terms of nutrient composition, *Spirulina* is a nutrient-rich supplement that contains all essential amino acids, vitamins and minerals. It also is a rich source of carotenoids and fatty acids, especially gamma-linolenic acid (GLA) that infer health benefits. After slaughter, subcutaneous adipose tissue (SAT) and *ld* samples were subjected to mRNA extraction and reverse transcription using quantitative polymerase chain reaction (RT-qPCR) to assess the mRNA expression levels of the *Aralkylamine N*-*acetyltransferase* (*AANAT*), *Adrenergic beta*-*3 receptor* (*ADRB3*), *B*-*cell translocation gene 2* (*BTG2*) and *Fatty acid synthase* (*FASN*) genes, which are associated with lipid metabolism.

**Results:**

Both low and high *Spirulina* supplementation levels strongly up-regulated the transcription of all the selected genes in both SAT and *ld* tissues (mostly in the subcutaneous adipose), but sheep breed and sex did not influence the gene expression patterns in these tissues.

**Conclusions:**

The evidence indicates that high *Spirulina* supplementation level resulted in a decrease in intramuscular fat content in Australian purebred and crossbred sheep due to the enhanced production of melatonin in sheep muscle tissues and strong up-regulation of mRNA expression of *BTG2* in SAT which negatively affected fat deposition. In contrast, low *Spirulina* supplementation level strongly up-regulated the *ADRB3* and *FASN* genes responsible for fat production. These findings are consistent with the observed phenotypic data suggesting that low *Spirulina* supplementation level can increase lamb production, with higher long-chain PUFA content.

## Background

Inclusion of marine supplements in the diet of sheep represents an effective nutritional strategy for altering meat production and enhancing polyunsaturated fatty acids (PUFA) [1,2]. *Spirulina* (*Arthrospira platensis*) is an edible blue-green microalga, highly nutritious supplement containing 60 to 70% protein and is a potential feed resource for many animal species. Research findings have linked *Spirulina* to an improvement in animal growth and nutritional product quality [2-4].

Meat with superior eating qualities and healthier nutritional composition commands a higher price that consumers are generally prepared to pay. Traditionally, the fat content of meat has been considered as an important source of essential fatty acids and as a calorie-dense nutrient [5]. Within the last decade, fats, and particularly fatty acids, have been increasingly recognised as major biological regulators of the quality of fresh meat and sensory value of meat products [6,7]. In addition, fatty acids can influence sterol metabolism, signal transduction, enzyme activities, cell proliferation, differentiation and receptor expression [5,6]. The cellular effect of fatty acids is related to the regulation of gene expression and subsequent downstream events, and omega-3 fatty acids are especially potent in affecting many of the metabolic pathways [6].

Fat deposition and composition can be altered by the animal’s genetic background and dietary supplements fed, thus indicating that both factors have significant influences on adipogenic and lipogenic metabolic pathways [8]. However, the molecular mechanisms underlying fat deposition and fatty acid composition in sheep are not yet fully understood. To our knowledge, there is only limited available information addressing the molecular adaptation of ovine tissues to supplementation with dietary marine ingredients that induce superior quality of meat production and PUFA enhancement.

This study aimed to investigate the changes in mRNA expression patterns of these key genes: *Aralkylamine N*-*acetyltransferase* (*AANAT*), *Adrenergic beta*-*3 receptor* (*ADRB3*), *B*-*cell translocation gene 2* (*BTG2*), and *Fatty acid synthase* (*FASN*) controlling lipid metabolism in subcutaneous adipose tissue (SAT) and *Longissimus dorsi* (*ld*) muscle of Australian purebred and crossbred lambs subjected to zero, low and high levels of dietary supplementation with *Spirulina*.

## Methods

### Experimental animals and data collection

The use of animals and procedures performed in this study were all approved by the University of Tasmania Animal Ethics Committee, and were conducted in accordance with the 1993 Tasmanian Animal Welfare Act and the 2004 Australian Code of Practice for the Care and Use of Animals for Scientific Purposes. The design of the experiment has been described previously [2]. Briefly, 24 crossbred lambs comprising 12 wethers and 12 ewes of purebred Merino, Black Suffolk x Merino, Dorset x Merino and White Suffolk x Merino crossbred lambs were randomly assigned into three supplementary treatment groups: the *control* group grazing without *Spirulina* (0%), *low* (100mL/head/day in the ratio of 1g of *Spirulina* powder:10mL of water or 10% wt/vol), and *high* (200mL/head/day in the ratio of 2g of *Spirulina* powder:10mL of water or 20% wt/vol) *Spirulina* supplementation levels. The *Spirulina* powder was purchased from a commercial producer in Darwin, Northern Territory, Australia (TAAU, NT, Aus). Lambs were daily supplemented according to their assigned *Spirulina* treatment group before being released into paddocks for grazing. All lambs had ad libitum access to clean drinking water and ryegrass pastures. Following slaughter after nine weeks of the feeding trial, approximately 10 g of subcutaneous adipose and *Longissimus dorsi* muscle tissues were collected from the carcass and frozen for subsequent RNA extraction and further analysis.

### RNA extraction and cDNA synthesis

Total RNA was isolated from the thawed frozen tissues using TRIzol® Plus RNA Purification Kit (Life Technologies Pty Ltd. Victoria, Australia). Homogenisation of the sample in TRIzol® reagent was performed using a tissue lyser (Qiagen Ltd., Crawley, UK). RNA was extracted using chloroform and precipitated using isopropanol. The quantity of total RNA extracted was assessed using the NanoDrop 8000 spectrophotometer (NanoDrop, Wilmington, DE). RNA quality was verified by ensuring that all RNA samples had an absorbance ratio (A260/280) between 1.8 and 2. RNA samples were treated with PureLink™DNase (Life Technologies Pty Ltd. Victoria, Australia) and purified using the RNeasy1 Mini Kit (Qiagen Ltd.). DNase-treated and purified total RNA was then reverse transcribed to cDNA with Mixed Oligo dT/Random Hexamer Primers using the Tetro cDNA Synthesis Kit (Bioline Pty Ltd. NSW, Australia) according to the manufacturer’s instructions and stored at −80°C for subsequent analyses.

### Primer design and reference gene selection

All candidate and reference gene primers used for gene expression (Table 1) were designed using the Primer3 web based software program (http://frodo.wi.mit.edu/primer3/), and obtained from a commercial supplier (GeneWorks Pty Ltd., SA, Australia). Primer specificity was checked using the Basic Local Alignment Search Tool (BLAST) at the National Center for Biotechnology Information (http://www.ncbi.nlm.nih.gov/BLAST/). All primers were validated using pooled cDNA samples. A standard curve was generated using serial dilutions of pooled cDNA. PCR products generated by amplification were sequenced to verify their primer specific identity (Beckman Coulter CEQ™ 8000 Series Genetic Analysis System, University Tasmania). To determine the relative gene expression levels, suitable and highly stable reference genes were required. A total of five reference genes were tested out of which two [*Ubiquitin C* (*UBC*) and *Peptidyl*-*prolyl cis*-*trans isomeraseA* (*PPIA*)] were finally chosen to normalise the gene expression data for *AANAT*, *BTG2*, *FASN* and *ADRB3* transcription levels in both tissues. The principle behind the selection of the two reference genes was their constant expression ratios across all samples. The gene expression stability (M-value) of the reference genes was calculated and validated using the *geNorm* software (version 3.5).

**Table 1:** **Primer pairs designed for real**-**time PCR** (**qPCR**)

^**a**^ **Gene symbol**	**qPCR primers**		^**b**^ **T**_**a**_	**Amplicon size** (**bp**)
	Forward Primer	Reverse Primer		
**AANAT**	ACTGACCTTCACGGAGATGC	TTCACTCATTCTCCCCGTTC	60	211
**ADRB3**	TCAGTAGGAAGCGGGTCGGG	GGCTGGGGAAGGGCAGAGTT	60	291
**BTG2**	CTGGAGGAGAACTGGCTGTC	AAAACAATGCCCAAGGTCTG	60	194
**FASN**	GTGTGGTACAGCCCCTCAAG	ACGCACCTGAATGACCACTT	60	110
**Reference genes**				
**UBC**	CGTCTTAGGGGTGGCTGTTA	AAATTGGGGTAAATGGCTAGA	60	90
**PPIA**	TCATTTGCACTGCCAAGACTG	TCATGCCCTCTTTCACTTTGC	60	72

^a^Aralkylamine N-acetyltransferase = AANAT, *β*3-adrenergic receptor = ADRB3, B-cell translocation gene 2 = BTG2, Fatty acid synthase = FASN, Ubiquitin C = UBC, Peptidyl-prolyl cis-trans isomeraseA = PPIA, ^*b*^Ta = Empirical annealing Temperature.

### Quantitative real time PCR (qPCR)

Following reverse transcription, cDNA quantity was determined and standardised to the required concentration for qPCR. Triplicate 20 μL reactions were carried out in a 72-well Rotor-Gene (QIAGEN GmbH, Hilden, Germany), containing 4 μL cDNA (50 ng), 10 μL 2× SensiFAST SYBR No-ROX Mix (Bioline Pty Ltd., NSW, Australia), 4.4 μL DEPC H_2_O, and 0.8 μL forward and reverse primers (100 fmol). Assays were performed using the Rotor-Gene 3000 (QIAGEN Pty Ltd., VIC, Australia) with the following cycling parameters: 95°C for 2 min polymerase activation; 40 cycles of 95°C for 5 s denaturation, 60°C for 10 s annealing and 72°C for 5 s extension. Gene expression levels were recorded as Ct values (i.e., the number of PCR cycles at which the fluorescence signal was detected above the threshold value) and all samples were run in triplicates. Amplification efficiencies were determined for all candidate and reference genes using the formula E = 10^(−1/slope), with the slope of the linear curve of cycle threshold (Ct) values plotted against the log dilution [9] (Higuchi et al., 1993). Primer concentrations were optimised for each gene and disassociation curves were examined for the presence of a single PCR product. The efficiency of the reaction was calculated using a 5-fold serial dilution of cDNA and generation of a standard curve. All PCR efficiency coefficients were between 1.7 and 1.8 and therefore deemed acceptable. The software package Rotor-Gene 3000 (version 6.0.16) (QIAGEN Pty Ltd., VIC, Australia) was used for efficiency correction of the raw Ct values. This process involved an inter-plate calibration based on a calibrator sample included on all plates, averaging of replicates, normalisation to the reference gene and the calculation of quantities relative to the highest Ct and log2 transformation of the expression values for all genes.

### Statistical analysis

A generalised linear model (GLM) (SAS Inst., NC) was used in computing the fixed effects of *Spirulina* supplementation level, sire breed and sex, and their interactions on mRNA expression level of *AANAT*, *ADRB3*, *BTG2* and *FASN* genes in subcutaneous adipose and muscle tissues. Separation of significant least squares means was tested at a minimum of 5% threshold using Tukey’s pairwise comparisons.

## Results

### *Spirulina* supplementation and phenotypic data

The candidate and reference gene primers used for the gene expression analysis are shown in Table 1. *Spirulina* supplementation enabled sheep to grow longer bodies (BL) than the control group (*P* < *0.015*) as portrayed in Table 2. Furthermore, lambs in the high *Spirulina* supplementation treatment group had greater body condition score (BCS) than the low and control treatment groups (*P* < *0.001*). It was observed that sheep receiving low *Spirulina* supplementation had the heaviest body weight (BWT) of 41.9 kg (*P* < *0.018*). However, no differences were observed between the high and control treatment groups. The phenotypic results are shown in Table 2.

**Table 2:** **Least square means** (**LSM**) **of average daily gain, body conformation, condition score** a**nd liveweight in** ***Spirulina*** **supplemented crossbred lambs**

	**Spirulina**			**Breed**				**Sex**		**P values**		
	Control	Low	High	BS	WS	D	M	W	E	Spirulina	Breed	Sex
**CG** **(cm)**	95.0	95.6	96.1	99.0^a^	94.4^b^	93.8^b^	95.0^b^	96.2^a^	94.9^b^	0.376^ns^	0.001^***^	0.034*
**WH** **(cm)**	62.9	62.7	63.1	63.6^a^	62.8^a^	63.5^a^	61.6^b^	63.4^a^	62.4^b^	0.669^ns^	0.001^***^	0.009**
**BL** **(cm)**	65.7^b^	66.6^a^	66.8^a^	68.8^a^	67.0^b^	66.9^b^	62.6^c^	66.5	66.2	0.015*	0.001^***^	0.269^ns^
**BCS** **(0–5)**	3.2^b^	3.3^b^	3.4^a^	3.7^a^	3.3^b^	3.2^b^	3.1^c^	3.3	3.3	0.001^***^	0.001^***^	0.346^ns^
**BWT** **(kg)**	40.6^b^	41.9^a^	40.8^b^	46.3^a^	42.9^b^	41.8^b^	33.5^c^	42.1^a^	40.1^b^	0.018**	0.001^***^	0.001^***^
**ADG** **(kg/d)**	0.1	0.2	0.1	0.1	0.2	0.2	0.1	0.1	0.1	0.759^ns^	0.502^ns^	0.605

*BS* = Black Suffolk, *WS* = White Suffolk, *D* = Dorset, *M* = Merino, *W* = Wethers, *E* = Ewes.

Column means within a fixed effect bearing different superscripts significantly differ (P < 0.05). Chest girth (*CG*), withers height (*WH*), body length (*BL*), body condition score (*BCS*), body weight (*BWT*), and average daily weight gain (*ADG*). Level of significance: ns not significant (P > 0.05), * significant (P < 0.05), ** highly significant (P < 0.01), and *** very highly significant (P < 0.001).

### Gene expression pattern

To determine the expression patterns of *AANAT*, *ADRB3*, *BTG2* and *FASN* genes, a panel of subcutaneous and adipose tissues was collected from 20 genetically divergent Australian purebred and crossbred sheep supplemented with either zero, low or high levels of *Spirulina*. The genes were investigated directly for mRNA expression by qRT-PCR molecular biology techniques. The qRT-PCR results were calibrated and normalized using two housekeeping genes (*UBC* and *PPIA*) and the qBase relative quantification excel application [10] for automated analysis.

### Gene expression in the subcutaneous adipose tissue

**Figure 1:**
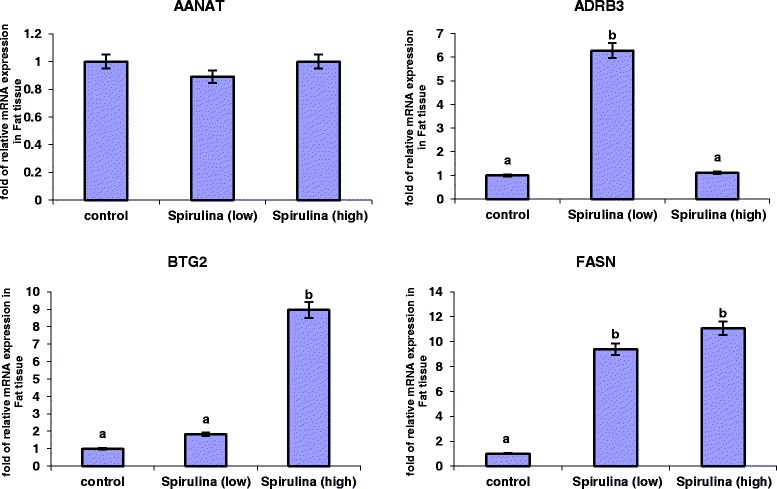
**Relative expression levels of AANAT**, **ADRB3**, **BTG2 and FASN genes in the subcutaneous adipose tissue of Australian purebred and crossbred sheep supplemented with 0**, **low and high levels of** ***Spirulina***. Least square means with different superscripts differ by at least *P* < 0.05.

The relative mRNA expression levels of *AANAT*, *ADRB3*, *BTG2* and *FASN* genes analysed in the subcutaneous adipose tissue (SAT) are presented in Figure 1. *ADRB3*, *BTG2* and *FASN* showed higher expression levels in tissues from sheep that received low or high levels of *Spirulina* supplementation, relative to the control. In contrast, *Spirulina* supplementation did not alter the mRNA expression of *AANAT* gene in SAT.

Ten percent dietary *Spirulina* supplementation up-regulated mRNA expression of the *ADRB*3 gene (*P* < 0.001), corresponding to a 5.27 folds change compared to the control treatment (Figure 1), whereas high *Spirulina* supplementation resulted in a down-regulation of *ADRB3* mRNA level, which is 5.08-folds lesser than the low *Spirulina* supplementation treatment and similar to the control group (1.19-folds higher). The mRNA expression level of *BTG*2 gene was significantly higher (*P* < 0.001) in tissues from sheep supplemented with high levels of *Spirulina* compared to either the 10% or control conditions (Figure 1). Low *Spirulina* dietary supplementation resulted in an up-regulation of *BTG*2 mRNA levels (0.83-folds) compared to the control, but this was not statistically significantly. However, high *Spirulina* supplementation resulted in a significantly higher expression level (7.95-folds increase) in SAT compared to control. A similar expression profile was observed for the *FASN* gene as dietary *Spirulina* up-regulated the mRNA expression levels in both the low and high supplementation groups; *FASN* expression increased 8.4-folds (*P* < 0.05) under the low supplementation treatment group and 10.06-folds under the high supplementation level, relative to control treatment (Figure 1).

### Gene expression in the *Longissimus dorsi* muscle

**Figure 2:**
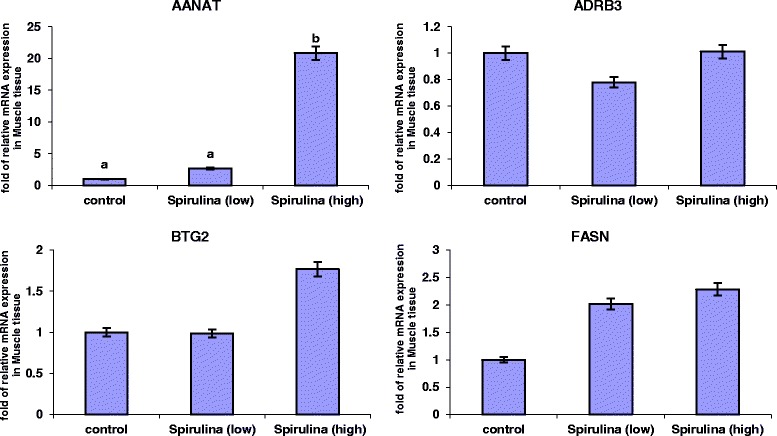
**Relative expression levels of AANAT**, **ADRB3**, **BTG2 and FASN genes in the** ***longissimus dorsi*** **muscle of Australian purebred and crossbred sheep supplemented with 0**, **low and high levels of** ***Spirulina*** **.** Least square means with different superscripts differ by at least *P* < 0.05.

The relative mRNA expression levels of the four genes in the *Longissimus dorsi* (*ld*) muscle tissue are presented in Figure 2. It was evident that under both the low and high dietary *Spirulina* supplementation groups, the mRNA expression levels of *AANAT* gene increased. However, this increase was only statistically significant in the high *Spirulina* supplementation treatment group, in which the *AANAT* mRNA expression level was 19.83-folds up-regulated compared to the control. *Spirulina* supplementation had no significant effect (*P* > 0.05) on the mRNA levels of *ADRB3*, *BTG2* and *FASN* genes (Figure 2). However, in the high *Spirulina* supplementation group, there was an up-regulation in the *BTG*2 (0.76-folds) and *FASN* (1.28-folds) mRNA levels compared to the control.

## Discussion

Fat and fatty acid deposition in animal tissues has been attributed to a complex regulation network of lipogenic genes, genetic differences, and diet. However, the molecular mechanisms underlying these systems remain to be fully understood and characterized. Therefore, understanding the factors affecting the depot-specific fat accretion and metabolism in sheep is of paramount importance. The present study addressed these aspects based on an experimental trial with four genetically diverse sheep breeds and three levels of protein-rich dietary supplementation with *Spirulina*. The results reported here indicate that genetic background (sire breed) and to a lesser extent sex, determined the mRNA expression levels of *AANAT*, *ADRB3*, *BTG2* and *FASN* genes. In order to elucidate the molecular mechanisms involved in this physiological process, these tissues and diet-specific variations are explained based on transcription levels of *AANAT*, *ADRB3*, *BTG2* and *FASN* genes.

### AANAT

The *AANAT* gene encodes an acetyltransferase superfamily protein [11]. This acetyltransferase is the penultimate enzyme in melatonin synthesis and controls the night/day rhythm in melatonin production in the vertebrate pineal gland. The AANAT protein catalyses the rate-limiting step in the synthesis of melatonin from serotonin [7]. Melatonin is essential for the function of the circadian clock that influences activity and sleep [11,12]. *AANAT* transcripts have been found to be differentially expressed in high vs. low omega-3 index (O3I) muscles, suggesting a role for melatonin in reducing oxidative damage, including that to PUFA [7,13].

The ability of melatonin to protect against lipid peroxidation has been repeatedly documented in many studies using animal and plant tissues [13]. Spanish scientists reported that melatonin consumption assists in the control of weight gain since it stimulates the appearance of brown fat (beige), a type of fat cell that burns calories instead of storing them [14]. Their research demonstrated that melatonin treatment not only induced browning of inguinal white adipose tissue in Zucker diabetic fatty rats, but also increased thermogenic activity [14]. Taken together, these findings highlight the anti-obesity effect of melatonin and explain its metabolic benefits of protecting against oxidative degradation of PUFA in the muscle tissue thereby producing higher O3I levels [13].

Herein, we report that *AANAT* transcription levels in the *Longissimus dorsi* muscle tissue are related to dietary *Spirulina* supplementation levels; high *Spirulina* supplementation resulted in a 7-fold and a 20-fold up-regulation of *AANAT* mRNA levels relative to the control and low *Spirulina* treatment groups, respectively. These findings suggest that sheep receiving the high *Spirulina* supplement may have lost body weight due to the enhanced production of melatonin in their muscle tissue, as observed in Table 2, and in accordance with phenotypic data from obese rats [14]. Therefore, a high level of *Spirulina* supplementation may be involved with, and results in, weight loss, which might occur through browning of the white adipose tissue, thus increasing the omega-3 levels in the fatty acid profile of the skeletal muscle tissue of sheep. However, further fatty acid analyses are required to confirm this hypothesis.

### ADRB3

*ADRB3* encodes a protein belonging to the adrenergic receptor group of G-protein coupled receptors [15]. ADRB3 is located mainly in adipose tissue and plays a key role in regulating mammalian energy storage and expenditure under the mediating effects of the sympathetic nervous system [16,17]. ADRB3 is the principal mediator of the lipolytic and thermogenic effect of high catecholamine (in particular norepinephrine), concentration in brown and white adipose tissues in rodents [16,18]. The primary role of the receptor is suggested to be the regulation of resting metabolic rate and lipolysis [19].

In a large number of studies, *ADRB3* gene expression has been shown to be correlated with obesity in both humans and other mammals [20,21]. Findings from various studies now provide a consistently clear picture of the important role of *ADRB3* in the regulation of lipid metabolism and make this protein an obvious target for drug discovery strategies designed to treat obesity [20].

Herein, we demonstrate that *ADRB3* transcription levels are significantly up-regulated in SAT under low dietary *Spirulina* supplementation, which is consistent with our observed phenotypic results in Table 2 that confirm weight gain in this group. The *ADRB3* mRNA expression levels under high *Spirulina* supplementation remained unchanged compared to the control group. One possible explanation for this observation is the negative correlation between protein accretion and fat deposition rates that had been exacerbated by high protein levels in feeds [2]. In addition, excess protein may become deaminated and lost in the urine or become broken down in the liver, which could result in fatty liver and ketosis [2]. Thus, we are able to speculate that the low level of *Spirulina* supplementation can be beneficial, giving higher production by fattening sheep in terms of early attainment of market weight. However, supplementing sheep with a higher dosage of *Spirulina* may result in lower efficiency in liver function and probably a decrease in total production. Further research into *ADRB3* transcription levels in sheep liver would allow greater insight into the underlying biological mechanism.

### BTG2

The mammalian *BTG2* gene belongs to the anti-proliferative (APRO) family of genes that regulate cell cycle progression in a variety of cell types [22,23]. *BTG2* is a prototypical member of the *BTG*/*TOB* family with anti-proliferative properties. The protein encoded by this gene controls cell cycle progression and proneural gene expression by acting as a transcription co-regulator that enhances or inhibits the activity of transcription factors [22,23].

In our study, it was apparent that *Spirulina* supplementation increased *BTG2* transcription levels in SAT. However, only tissues from sheep receiving a high level of *Spirulina* supplementation significantly over-expressed the *BTG2* gene. A number of studies have demonstrated that the *BTG2* gene has a potential role in muscle fibre size, intramuscular fat deposition and weight loss [22,23]. Herein, we suggest that the weight loss experienced by the high *Spirulina* supplementation group may be attributable to a decline of preadipocyte proliferation, an increase in energy expenditure and a decline in energy uptake in adipocytes, which may be caused by an increase in *BTG2* expression.

### FASN

*FASN* encodes a multifunctional enzyme that catalyses fatty acid synthesis [24]. FASN is considered as a fundamental enzyme in *de novo* lipogenesis in mammals and its main function is to catalyse the synthesis of palmitate from acetyl-CoA and malonyl-CoA, in the presence of NADPH, into long-chain saturated fatty acids (LC-SFA) [24,25]. It has been shown that the *FASN* gene contributes to the regulation of body weight in humans, which results in the development of obesity [24,26].

We demonstrate herein, that both the low and high levels of *Spirulina* supplementation increased the transcription levels of *FASN* in both SAT and muscle tissues, which may have assisted these sheep in gaining weight.

Our results demonstrate that supplementing sheep with low levels of *Spirulina* can increase lamb production by increasing the transcription level of *ADRB3* and *FASN* genes in SAT, and might also beneficially alter the fatty acid profile by reducing the oxidation of PUFA in skeletal muscle.

## Conclusions

The results presented herein suggest that the mRNA levels of *AANAT*, *ADRB3*, *BTG2* and *FASN* genes in the SAT and *Longissimus dorsi* muscle tissues are mainly influenced by dietary *Spirulina* level, whereas the individual effects of breed and sex, and their combined effects with diet, are not associated with the mRNA expression levels of the above genes. Taken together, our results show that lipid metabolism in SAT is more sensitive to dietary supplementation than in the muscle. These findings provide evidence to support a low to intermediate level of dietary protein supplementation for achieving optimal increase in the omega-3 and 6 contents of red meat in Australian purebred and crossbred sheep.
